# Synthesis and Antitumor Activity of Erlotinib Derivatives Linked With 1,2,3-Triazole

**DOI:** 10.3389/fphar.2021.793905

**Published:** 2022-01-17

**Authors:** Peng Deng, Ge Sun, Jie Zhao, Kaitai Yao, Miaomiao Yuan, Lizeng Peng, Longfei Mao

**Affiliations:** ^1^ Key Laboratory of Agro-Products Processing Technology of Shandong Province, Key Laboratory of Novel Food Resources Processing Ministry of Agriculture, Institute of Agro-Food Science and Technology Shandong Academy of Agricultural Sciences, Jinan, China; ^2^ School of Basic Medical Sciences, Cancer Research Institute, Southern Medical University, Guangzhou, China; ^3^ Henan Engineering Research Center of Chiral Hydroxyl Pharmaceutical, School of Chemistry and Chemical Engineering, Henan Normal University, Xinxiang, China; ^4^ The Eighth Affiliated Hospital, Sun Yat-sen University, Shenzhen, China

**Keywords:** EGFR, erlotinib, 1,2,3-triazole, HeLa, antitumor activity

## Abstract

Cervical cancer is one of the most important cause of cancer-related death and presents a major public health problem in many countries. To search for more novel antitumor agents against cervical cancer, 14 erlotinib-linked 1,2,3-triazole compounds were designed, synthesized, and evaluated for their anti-tumor activity. The compounds were confirmed by ^1^H NMR, ^13^C NMR, and high-resolution mass spectra (HR MS). Antitumor activity assay results indicated that six of those compounds have remarkable inhibitory activity against human cervical cancer HeLa cells *in vitro*, among which compound 4m was the most potent with IC_50_ of 3.79 μM, and compounds 4k, 4i, 4l, 4d, and 4n also demonstrated remarkable antitumor activity with IC_50_ of 3.79, 4.16, 4.36, 7.02, and 8.21 μM. We found three of the most potent compounds 4d, 4k, and 4l induced potent apoptosis and cell cycle arrest in HeLa cells, and compounds 4d and 4l significantly restrained the cell colony formation and showed moderate epidermal growth factor receptor (EGFR) inhibitory activity with IC_50_ of 13.01 and 1.76 μM. Therefore, these experiments indicate that these erlotinib-linked 1,2,3-triazole compounds are potential to act as effective anticancer agents against cervical cancer.

## Introduction

Among women, cervical cancer ranks fourth for both incidence (6.6%) and mortality (7.5%) and is one of the most important cause of cancer-related death (Bray et al., 2018). Even if human papilloma virus (HPV) vaccination were approved and early screening efforts have been made, cervical cancer still represents a major public health problem in many countries due to increased percentage of locoregional and distant recurrences in advanced-inoperable cervical cancer. Furthermore, recurrent cervical cancer is not amenable to radical treatment, and *de novo* metastatic disease are considered incurable with poor prognosis (Liontos et al., 2019; Hill 2020). Thus, new active anticancer agents and their optimal combinations treatment are desperately needed.

**FIGURE 1:**
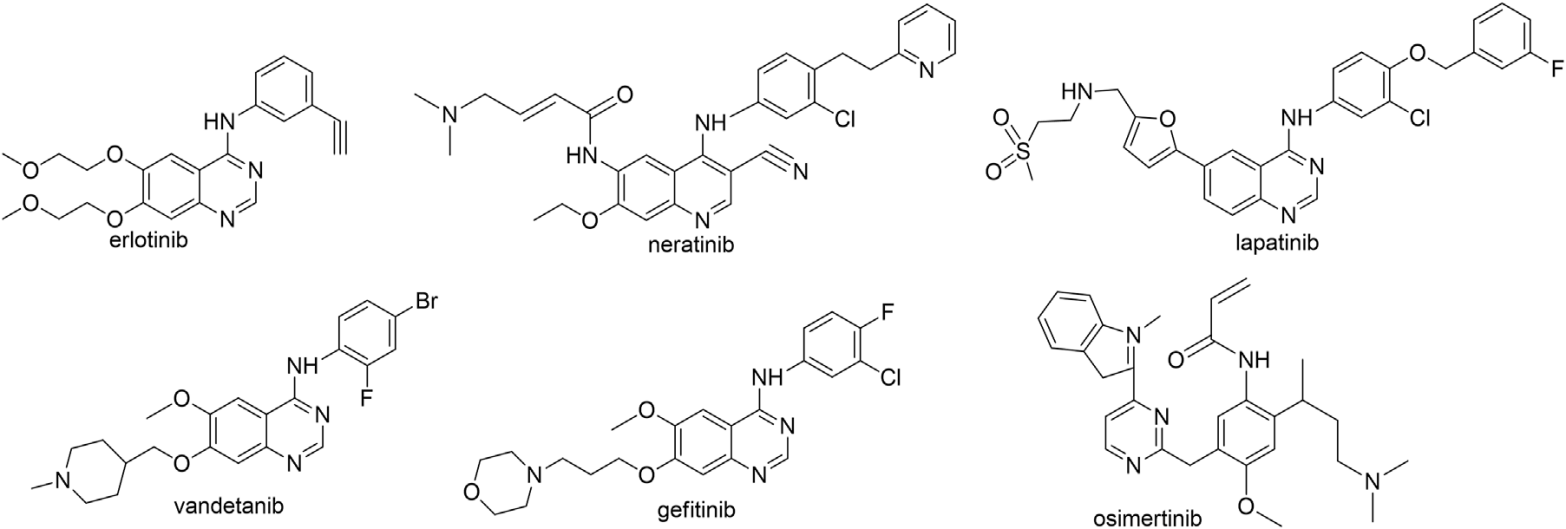
The reported EGFR inhibitors.

Epidermal growth factor receptor (EGFR) is a protein tyrosine kinase transmembrane receptor encoded by proto-oncogene HER-1 (Roskoski 2014) and is overexpressed in a variety of cancers such as breast, cervical, liver, and non-small cell lung cancers. Quinazoline-based EGFR kinase inhibitors such as erlotinib (Schettino et al., 2008), neratinib, lapatinib (Tan et al., 2015), vandetanib (Yin et al., 2019), gefitinib (Cohen et al., 2003; Zhang et al., 2017), and osimertinib (Jänne et al., 2015; Qin et al., 2016) (Figure 1) are the major EGFR inhibitors used clinically. These EGFR kinase inhibitors and their derivates have been well studied and proved to be effective on various cancers including cervical cancer cells (Bhatia et al., 2020). Erlotinib is a classical EGFR inhibitor and was approved in 2003 for the treatment of advanced NSCLC that deteriorates after traditional chemotherapy (Mathew et al., 2015). Compared with traditional chemotherapy drugs, erlotinib improved the median survival rate from 4 months to more than 40 months and performs better in terms of progression-free survival rate, quality of life, and tolerability (Hirsch et al., 2017).

**FIGURE 2:**
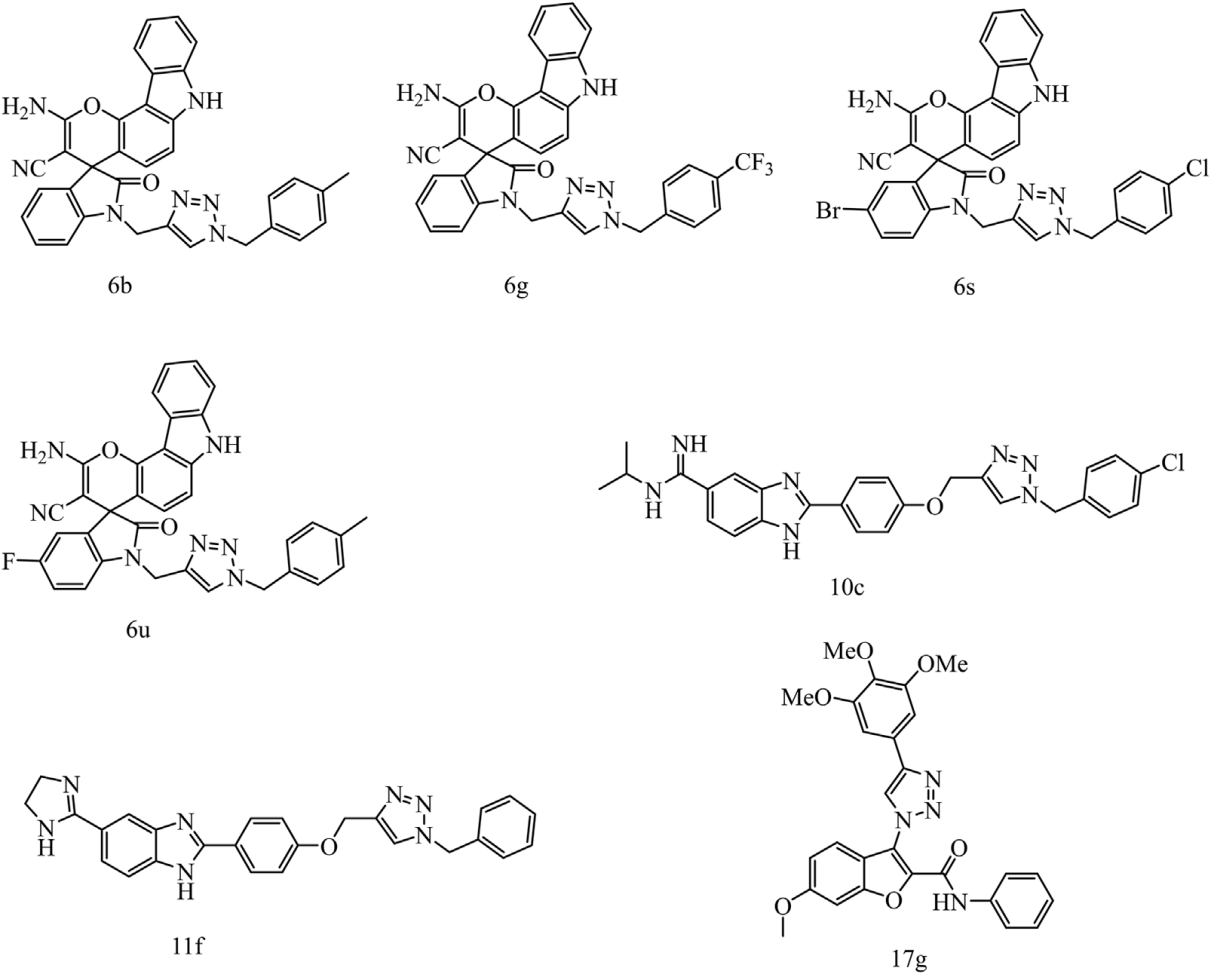
The structures of compounds containing 1,2,3-triazole unit with anticancer activities against HeLa cells.

1,2,3-Triazole is one of the important N-heterocyclic building blocks, and it plays a significant role in many compounds containing 1,2,3-triazole unit, which show good inhibitory effect against inflammation, cancer, and microbes (Mao L. et al., 2020). Moreover, CuAAC reaction (Safavi et al., 2018; Maddili et al., 2018; Saeedi et al., 2019), a convenient and regiospecific method of constructing 1,4-disubstituted triazoles (Thomopoulou et al., 2015), has aroused great interest and has been widely used in drug discovery (Hong et al., 2010). Our previous work revealed that compounds containing 1,2,3-triazole moiety exhibited good bioactivities such as antitumor or antibacterial activity (Mao LF. et al., 2020). In addition, a lot of studies demonstrated that compounds containing 1,2,3-triazole unit have potent anticancer activities against cervical cancer HeLa cells. In a study of Sarkar *et al.*, a series of chromenocarbazole tethered 1,2,3-triazoles were designed and synthesized by Click chemistry based one-pot five-component reaction. Compounds 6b, 6g, 6s, and 6u showed excellent antiproliferative activity (IC_50_ = 4.05, 3.54, 3.83, and 3.35 μM, respectively) in HeLa cells (Figure 2) (Chavan et al., 2019). In another study, Raic-Malic et al. synthesized a series of novel amidino 2-substituted benzimidazoles linked to 1,4-disubstituted 1,2,3-triazoles, and two of the new compounds 10c and 11f show potent antiproliferation activities against HeLa cells (IC_50_ = 17.53 and 6.63 μM, respectively), which could be attributed to induction of apoptosis and primary necrosis. Besides, Chen et al. synthesized a series of 1-(benzofuran-3-yl)-4-(3,4,5-trimethoxyphenyl)-1H-1,2,3-triazole derivatives and determined their antiproliferative activities against HCT116, HeLa, HepG2, and A549 cells, which could be associated with tubulin polymerization inhibitory activities. One of these compounds, 6-methoxy-N-phenyl-3-[4-(3,4,5-trimethoxyphenyl)-1H-1,2,3-triazol-1-yl] benzofuran-2 carboxamide (17 g) exhibited potent antiproliferative activities against HeLa cells, with IC_50_ values of 0.73 ± 0.67 μM (Qi et al., 2020).

In order to search new molecules with antitumor activity, we substituted 1,2,3-triazole unit for alkynyl in the structure of erlotinib *via* CuAAC reaction to obtain fourteen 1,2,3-triazole derivatives that have never been reported in literatures, and their *in vitro* inhibition of HeLa cell activity were also screened.

## Chemistry

**FIGURE 3:**
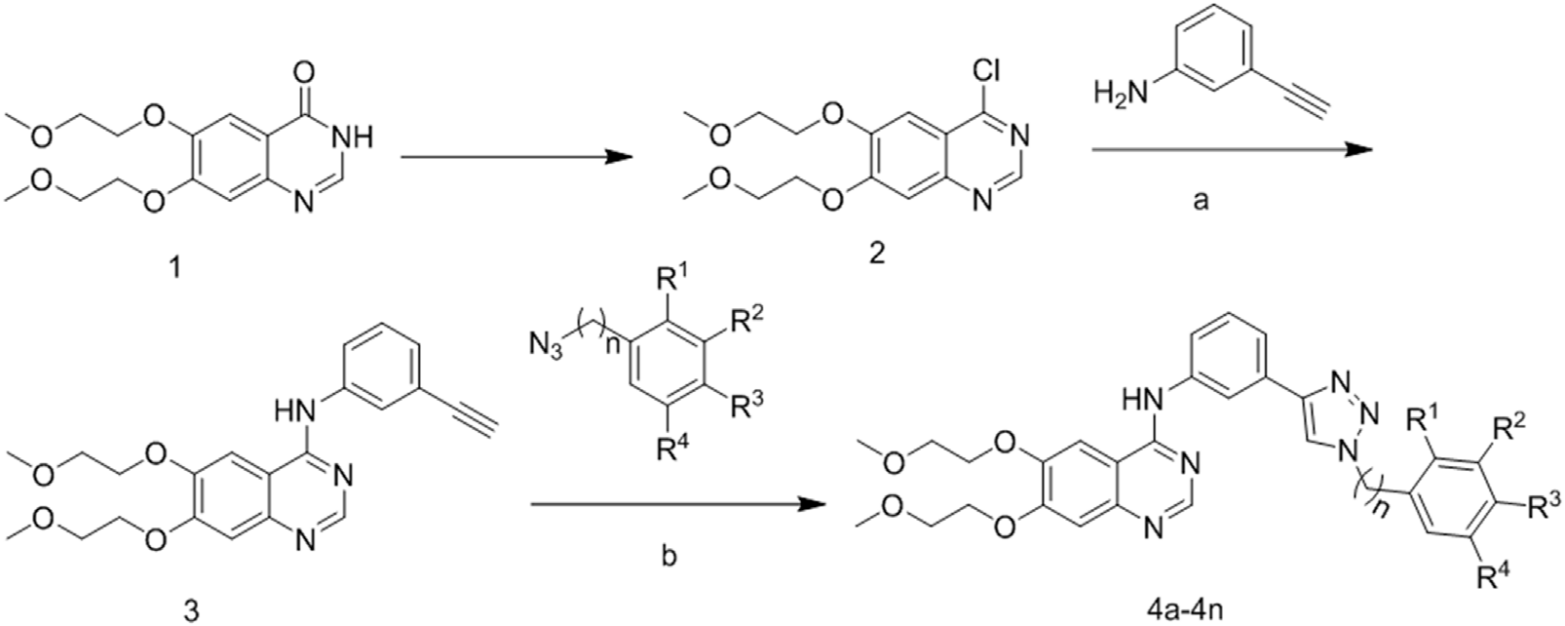
Reagents and conditions. **(A)** 85°C, isopropanol; **(B)** 60°C, copper sulfate pentahydrate; sodium ascorbate.

The synthetic strategy for the preparation of the target compounds is illustrated in Figure 3. The chlorination of hydroxyl group from compound **1** with SOCl_2_ produced 4-chloro-6,7-bis(2-methoxyethoxy) quinazoline (compound 2). Compound 2 reacted with 3-ethynylaniline through nucleophile substitution reaction to produce erlotinib (compound 3). Copper(I)-catalyzed azide–alkyne cycloaddition between erlotinib and different azido compounds afforded the target compounds **4a**–**4n**. The reaction conditions of the steps were convenient and easy to control. The structures of some key intermediates and all target compounds were confirmed by nuclear magnetic resonance (^1^H NMR and ^13^C NMR) and high-resolution mass spectrometry (HR MS).

## Results and Discussion

### Inhibition of HeLa Cells by Erlotinib-1,2,3-Triazole Derivatives

As demonstrated in Table 1, antitumor activity showed that six compounds exhibited higher antitumor activity than erlotinib, such as **4d** (IC_50_ = 7.02 μM), **4i** (IC_50_ = 4.36 μM), **4k** (IC_50_ = 4.16 μM), **4l** (IC_50_ = 4.51 μM), **4m** (IC_50_ = 3.79 μM), and **4n** (IC_50_ = 8.21 μM), indicating that the introduction of triazole enhanced the antitumor activity of HeLa.

**TABLE 1:** Antitumor inhibitory activity of compounds **4a**-**4n**.

Compd no	n	R1	R2	R3	R4	IC_50_ (μM)
						HeLa
4a	1	H	H	H	H	>50
4b	1	I	H	H	H	11.50 ± 1.69
4c	1	Br	H	H	H	21.31 ± 7.79
**4d**	1	**H**	**Br**	**H**	**Br**	**7.02 ± 0.04**
4e	1	H	OCH_3_	H	H	>50
4f	0	F	H	H	H	30.66 ± 1.83
4g	0	H	H	F	H	9.05 ± 0.53
4h	0	Cl	H	H	H	8.85 ± 0.56
4i	0	Br	H	H	H	4.36 ± 0.15
4j	0	H	H	Br	H	12.22 ± 1.00
**4k**	0	**OCH**_**3**_	**H**	**H**	**H**	**4.16 ± 0.48**
**4l**	0	**H**	**H**	**CH**_**3**_	**H**	**4.51 ± 0.08**
4m	0	H	NO_2_	H	H	3.79 ± 0.1
4n	0	H	OCH_2_CH_3_	H	H	8.21 ± 1.05
Erlotinib	—	—	—	—	—	39.50 ± 3.34

a IC_50_, compound concentration required to inhibit tumor cell proliferation by 50% (mean ± SD, *n* = 3).

IC50, (μM): 1–10 (very strong), 11–25 (strong), 26–50 (moderate), 51–100 (weak), above 100 (non-cytotoxic).

Bold values represents the compounds tested in the later part of this manuscript.

### Cell Apoptosis Assay

**FIGURE 4:**
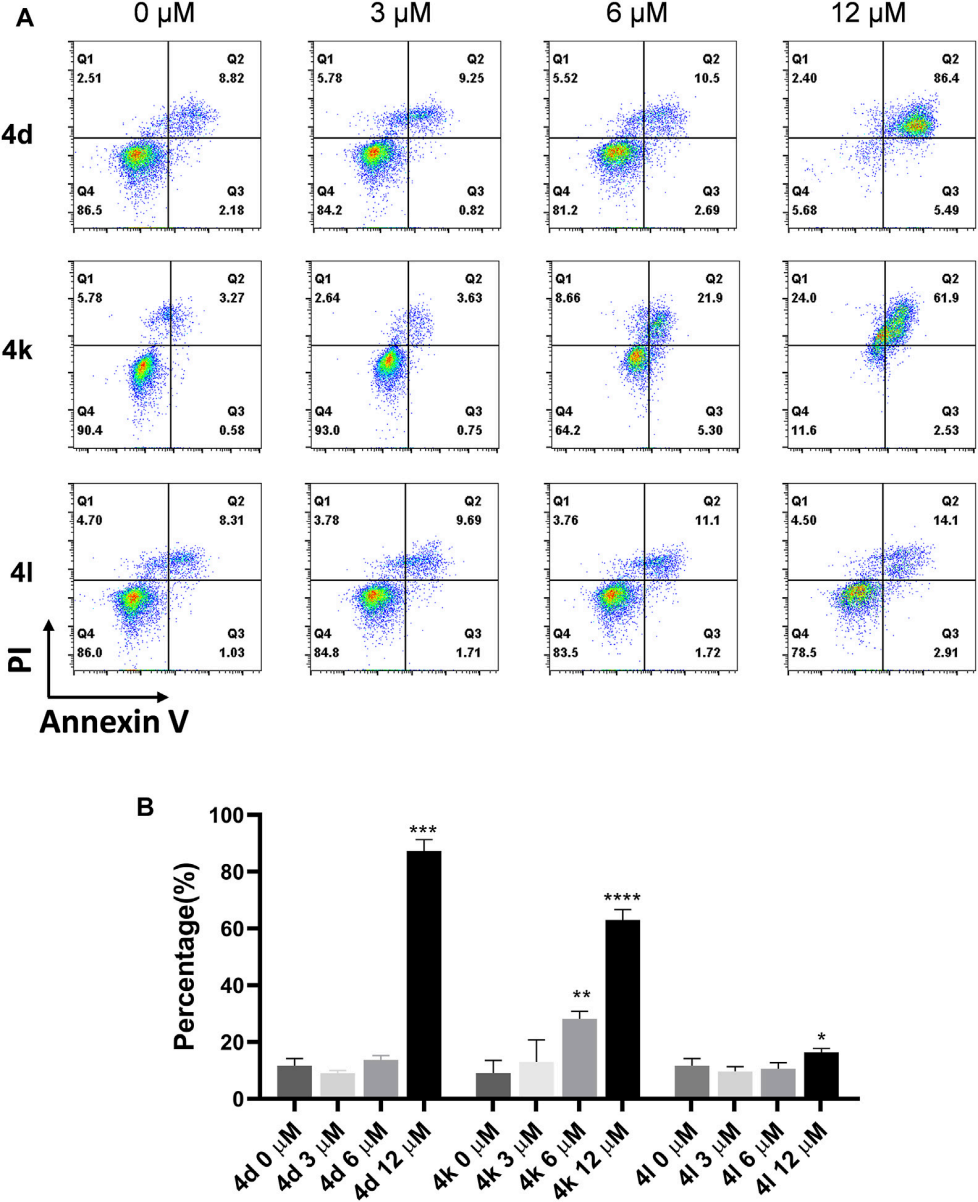
Compound 4d, 4k, and 4l induced apoptosis of HeLa cells. **(A)** HeLa cells were exposed to compound 4n for 72 h. **(B)** Flow cytometry analysis data from three independent experiments were summarized and shown. 0 μM, negative control. *p value < 0.05, **p-value < 0.01, ***p-value < 0.001 and ****p-value < 0.0001 (*t*-test).

To clarify whether the antiproliferative efficacy of the new compounds was associated with apoptosis, HeLa cells were treated with compounds 4d, 4k, and 4l (3, 6, and 12 μM) for 72 h, respectively, and then detected by flow cytometry. As shown in Figure 4, high concentration (12 μM) of 4d and 4l induced significant cell apoptosis in HeLa cells with percentages of 87.28% and 16.36%, respectively, and 6 and 12 μM of 4d induced significant cell apoptosis in HeLa cells with percentages of 28.21% and 62.96%, respectively. Therefore, 4d and 4k showed more robust efficacy in inducing HeLa cell apoptosis than 4l.

### Cell Cycle Assay

**FIGURE 5:**
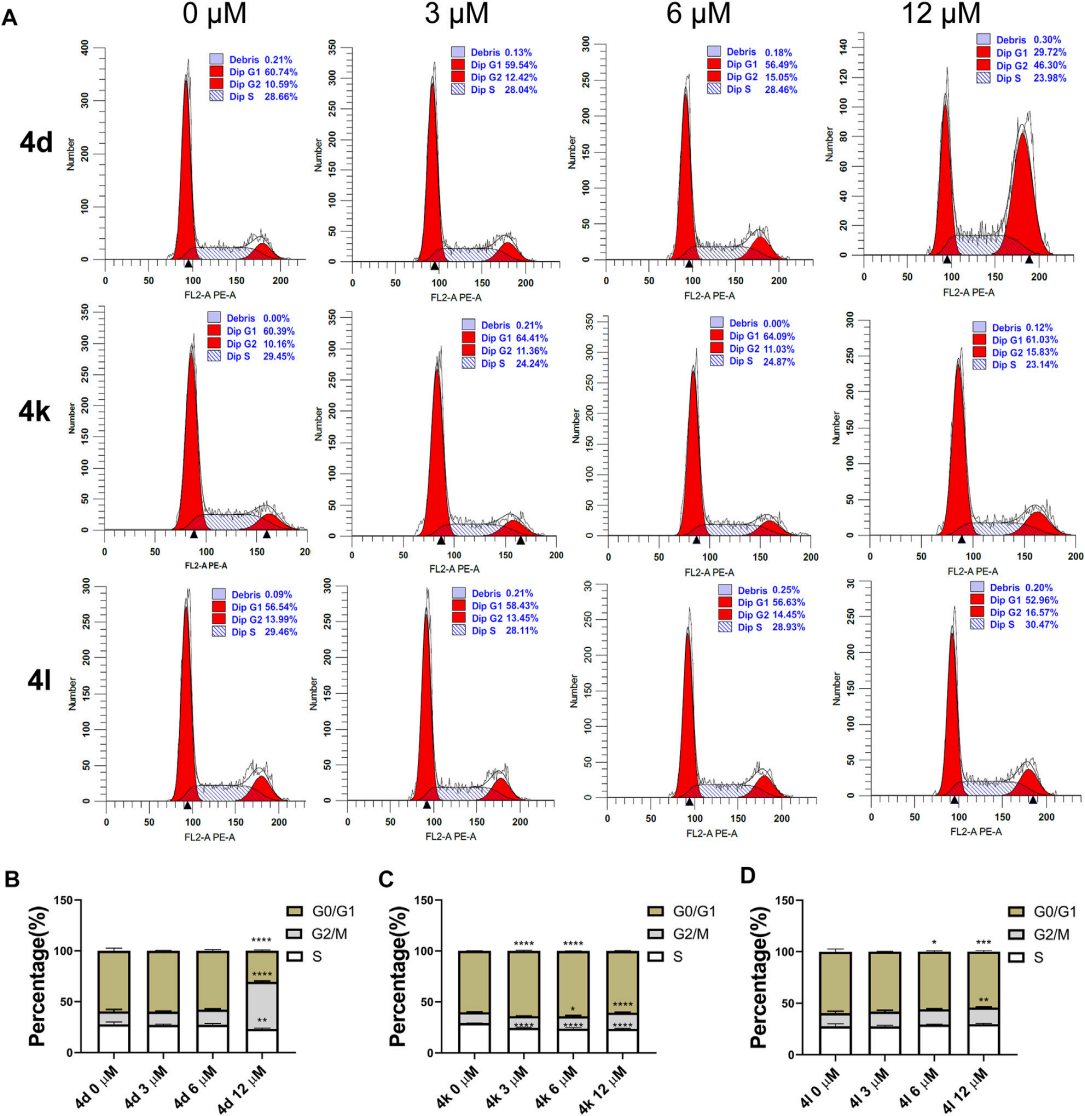
Cell cycle arrests induced by compounds 4d, 4k, and 4l in HeLa cells. **(A)** HeLa cells were exposed to compound 4d, 4k, and 4l for 48 h at various concentrations, respectively. The cell cycle and the proportions of each phase were detected through flow cytometry. **(B–D)** The percentages are summarized and shown. This experiment was repeated three times. 0 μM, negative control. *p < 0.05, **p < 0.01, ***l < 0.001 and ****p-value < 0.0001 (One-way ANOVA).

To investigate the effects of compounds 4d, 4k, and 4l on various phases of cell cycle, HeLa cells were treated with various concentrations of compounds 4d, 4k, and 4l for 48 h. As shown in Figure 5, the results of flow cytometry indicated that 4d and 4L induced higher percentages of HeLa cells in G2/M phrases at the concentration of 12 μM. However, 3 μM of compound 4k arrested HeLa cells at G0/G1 phrases, 12 μM of compounds 4k arrested HeLa cells at G2/M phrases, but 6 μM of compounds 4k arrested HeLa cells at both G0/G1 and G2/M. The results confirmed that compounds 4d, 4k, and 4l can inhibit the proliferation of HeLa cells through cell cycle arrests.

### Colony Formation Assay

**FIGURE 6:**
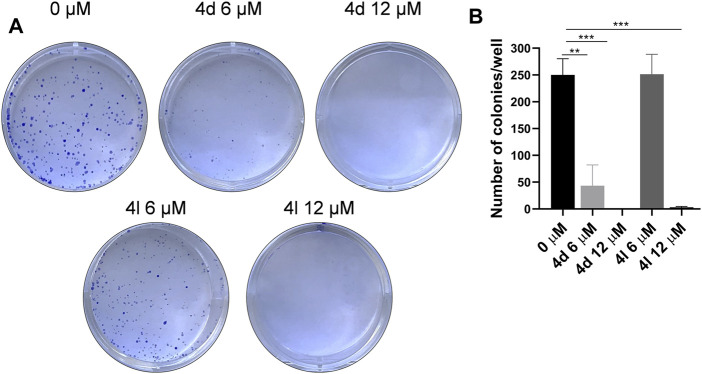
Compounds 4d and 4l inhibited cell survival in the colony formation assay. **(A)** Colony formation assay results. Images represents wells of HeLa cells exposed to each treatment. **(B)** Quantitative analysis of colony formation assay. The experiments were preformed three times. *p > 0.05, **p > 0.01, ***p > 0.001 (*t*-test).

To further evaluate the antiproliferation activities of these new compounds against HeLa cells, colony formation assay was performed. As the results demonstrated in Figure 6, compounds 4d and 4l entirely inhibited the cell colony formation at the concentration of 12 μM, but compound 4d can decrease the number of colonies at the concentration of 6 μM as well, suggesting that these compounds impeded the survival and proliferation of HeLa cells.

### EGFR Inhibition Study

To clarify whether the antiproliferative efficacy of the new compounds was associated with EGFR inhibitory activities, compound 4d, 4k, and 4l were assayed for their activities to inhibit EGFR tyrosine phosphorylation *in vitro* using ELISA. Erlotinib served as a positive control. The results were shown as IC_50_ values in Table 2. Under these conditions, the IC_50_ of erlotinib was 4.8 nM, which was similar to previously reported values (Moyer et al., 1997; Akita and Sliwkowski 2003) (IC_50_ = 2 nM). As illustrated in Table 2, compounds 4d and 4l showed more ability to inhibit EGFR tyrosine phosphorylation with the IC_50_ values of 13.01 and 1.76 μM.

**TABLE 2:** EGFR inhibitory activities of the derivatives.

Compd no	N	R^1^	*R* ^2^	R^3^	R^4^	IC_50_ (μM) EGFR
4d	1	H	Br	H	Br	13.01
4k	0	OCH_3_	H	H	H	49.39
4l	0	H	H	CH_3_	H	1.76
Erlotinib	—	—	—	—	—	0.0048

Kinase inhibitory activities of the compounds were evaluated using the enzyme-linked immunosorbent assay (ELISA).

## Conclusion

In summary, a series of erlotinib derivatives containing 1,2,3-triazole rings were prepared and evaluated for the antiproliferative activities against HeLa cells. Some of the compounds exhibited better antiproliferation activities than the parent erlotinib. Besides, **4d** (IC_50_ = 7.02 μM), **4k** (IC_50_ = 4.16 μM), and **4l** (IC_50_ = 4.51 μM) were demonstrated to induce apoptosis and cell cycle arrests in HeLa cells. In addition, 4d and 4l proved to impeded the survival and proliferation of HeLa cells by colony formation assay and showed considerable EGFR inhibitory activity. Therefore, these erlotinib-1,2,3-triazole compounds with potent anticancer activities may serve as novel antitumor agents against cervical cancer, and additional mechanisms merit further investigation.

## Experimental Protocols

### Materials and Chemistry

Erlotinib-1,2,3-triazole derivatives were in-house synthesized. All compounds were purchased from Aladdin’s reagent (China). All reagents and solvents obtained from commercially available source were used without further treatment.^1^H NMR and ^13^C NMR spectra were acquired in DMSO-d_6_ solution with a Bruker 600 spectrometer. Chemical shifts (d) were given in parts per million with tetramethylsilane as internal reference, and coupling constants were expressed in Hertz. HR MS measurements were carried out using a Bruker MicrOTOF-Q II mass spectrometer. HeLa cell line, Dulbecco’s modified Eagle’s medium (DMEM) medium, and fetal bovine serum were purchased from ATCC (Virginia, United States).

### General Procedure for the Synthesis of Analogues 4a–4n

#### General Procedure for Preparation of Compound 2

Compound **1** (30g, 0.1 mol) was added to a solution of dimethylformamide (DMF) (5 g) and thionylchloride (400 ml) at room temperature; then, the suspension was raised to 80°C and stirred for 3 h under nitrogen. After that, the mixture was cooled to 0°C–10°C, adjusted to pH7–9 with aqueous NaOH and extracted with dichloromethane (200 ml). The organic was washed with aqueous NaCl and evaporated to yield compound **2** as a tan solid.

#### General Procedure for Preparation of Erlotinib (Compound 3)

3-Aminophenylacetylene (1.2 g, 0.01 mol) was added to a suspension of compound **2** (3 g, 0.01 mol) and isopropanol alcohol (50 ml); then, the mixture was stirred at 85°C for 6 h under nitrogen. Solid gradually separated, and the reaction was monitored with thin-layer chromatography (TLC). After the completion of the reaction, the reaction mixture was transferred to ice water and stirred for half an hour. The solid was collected by filtration and was washed twice with isopropanol (30 ml) to give 2.1 g of erlotinib. ^1^H NMR (600 MHz, DMSO-d_6_): *δ* 9.48 (s, 1H), 8.51 (s, 1H), 8.00 (s, 1H), 7.91 (d, J = 9.5 Hz, 1H), 7.87 (s, 1H), 7.41 (t, J = 7.9 Hz, 1H), 7.27 – 7.17 (m, 2H), 4.30 (d, J = 15.1 Hz, 4H), 4.21 (s, 1H), 3.78 (d, J = 31.1 Hz, 4H), 3.38 (s, 3H), 3.36 (s, 3H);^13^C NMR (150Hz, DMSO-d_6_): 156.59, 154.15, 153.27, 148.61, 147.49, 140.28, 129.37, 126.81, 125.21, 123.02, 122.21, 109.39, 108.69, 103.65, 83.97, 81.03, 70.59, 70.52, 68.85, 68.52, 58.87, 58.82; HRMS (ESI) m/z: calcd for C_22_H_23_O_4_N_3_Na[M + Na]^+^416.1581, found 416.1585.

#### General Procedure for Preparation of Compounds 4a–4n

Erlotinib (1.0 mmol) and aryl-azido (1.2 mmol)were added to a mixed solvent (water:*tert*-butanol = 2:1, 30 ml). Cuprous iodide (0.1 mmol) was added to the mixture, and the reaction was stirred at 80°C. After completion of the reaction (monitored by TLC), the mixture was extracted with dichloromethane (20 ml × 3).The combined organic phase was washed successively with water and brine, then dried with anhydrous sodium sulfate and desolventized. The residue was purified by column chromatography (CH_2_Cl_2_/MeOH = 30:1) to obtain the desired compound **4** as a crystalline powder.

[3-(1-Benzyl-1H-[1,2,3]triazol-4-yl)-phenyl]-[6,7-bis-(2-methoxy-ethoxy)-quinazolin-4-yl]-amine (4a). m.p.89-92°C; ^1^H NMR (600 MHz, DMSO-d_6_): *δ* 9.56 (s, 1H), 8.67 (s, 1H), 8.49 (s, 1H), 8.27 (s, 1H), 7.95 – 7.86 (m, 2H), 7.56 (d, J = 7.7 Hz, 1H), 7.51 – 7.28 (m, 6H), 7.24 (s, 1H), 5.67 (s, 2H), 4.31 (d, *J* = 21.7 Hz, 4H), 3.78 (d, *J* = 32.4 Hz, 4H), 3.39 (s, 3H), 3.36 (s, 3H). ^13^C NMR (150Hz, DMSO-d_6_): 156.83, 154.06, 153.40, 148.56, 147.44, 147.12, 140.52, 136.50, 131.39, 129.48, 129.30, 128.66, 128.42, 122.28, 122.12, 120.79, 119.23, 109.43, 108.68, 103.69, 70.60, 70.54, 68.83, 68.51, 58.88, 58.82, 53.53; HR MS (ESI) m/z: calcd for C_29_H_30_O_4_N_6_Na [M + Na]^+^ 549.2221, found 549.2231.

[6,7-Bis-(2-methoxy-ethoxy)-quinazolin-4-yl]-{3-[1-(2-iodo-benzyl)-1H-[1,2,3]triazol-4-yl]-phenyl}-amine (4b). m.p.93-96°C; ^1^H NMR (600 MHz, DMSO-d_6_): *δ* 9.63 (s, 1H), 8.64 (s, 1H), 8.54 (s, 1H), 8.32 (s, 1H), 8.06 – 7.90 (m, 3H), 7.63 (d, *J* = 7.7 Hz, 1H), 7.50 (dd, *J* = 16.4, 8.0 Hz, 2H), 7.28 (s, 1H), 7.20 (dd, *J* = 11.8, 7.6 Hz, 2H), 5.75 (s, 2H), 4.35 (d, *J* = 20.8 Hz, 4H), 3.83 (d, *J* = 31.5 Hz, 4H), 3.43 (s, 3H), 3.41 (s, 3H). ^13^C NMR (150Hz, DMSO-d_6_): 156.86, 154.09, 153.34, 148.57, 147.30, 146.93, 140.50, 140.00, 138.36, 131.32, 130.80, 130.17, 129.49, 129.38, 122.52, 122.38, 120.88, 119.30, 109.42, 108.58, 103.72, 99.70, 70.59, 70.54, 68.84, 68.52, 58.88, 58.83, 58.03; HR MS (ESI) m/z: calcd for C_29_H_29_O_4_N_6_INa [M + Na]^+^ 675.1187, found 675.1196.

[6,7-Bis-(2-methoxy-ethoxy)-quinazolin-4-yl]-{3-[1-(2-bromo-benzyl)-1H-[1,2,3]triazol-4-yl]-phenyl}-amine (4c). m.p.94-97°C; ^1^H NMR (600 MHz, DMSO-d_6_): *δ* 9.60 (s, 1H), 8.63 (s, 1H), 8.50 (s, 1H), 8.27 (s, 1H), 7.98 – 7.84 (m, 2H), 7.72 (d, *J* = 7.9 Hz, 1H), 7.58 (d, *J* = 7.7 Hz, 1H), 7.46 (dt, *J* = 11.5, 7.7 Hz, 2H), 7.34 (t, J = 8.3 Hz, 1H), 7.29 – 7.16 (m, 2H), 5.76 (s, 2H), 4.31 (d, *J* = 20.5 Hz, 4H), 3.78 (d, *J* = 31.6 Hz, 4H), 3.38 (s, 3H), 3.36 (s, 3H). ^13^C NMR (150Hz, DMSO-d_6_): 156.88, 154.10, 153.31, 148.58, 147.22, 146.92, 140.47, 135.28, 133.42, 131.32, 131.00, 129.94, 129.50, 128.83, 123.37, 122.53, 122.41, 120.91, 119.33, 109.41, 108.53, 103.72, 87.36, 70.60, 70.53, 68.84, 68.52, 58.88, 58.83, 53.63; HR MS (ESI) m/z: calcd for C_29_H_29_O_4_N_6_BrNa [M + Na]^+^ 627.1331, found 627.1336.

[6,7-Bis-(2-methoxy-ethoxy)-quinazolin-4-yl]-{3-[1-(3,5-dibromo-benzyl)-1H-[1,2,3]triazol-4-yl]-phenyl}-amine (4d). m.p.102-105°C; ^1^H NMR (600 MHz, DMSO-d_6_): *δ* 9.58 (s, 1H), 8.72 (s, 1H), 8.49 (s, 1H), 8.28 (s, 1H), 7.99 – 7.89 (m, 2H), 7.86 (s, 1H), 7.64 (s, 2H), 7.57 (d, *J* = 7.6 Hz, 1H), 7.47 (t, *J* = 7.9 Hz, 1H), 7.24 (s, 1H), 5.70 (s, 2H), 4.35 – 4.26 (m, 4H), 3.83 – 3.73 (m, 4H), 3.39 (s, 3H), 3.36 (s, 3H); ^13^C NMR (150Hz, DMSO-d_6_): 156.84, 154.06, 153.39, 148.56, 147.45, 147.22, 140.83, 140.56, 133.73, 131.21, 130.67, 129.51, 123.23, 122.41, 120.83, 119.30, 109.44, 108.68, 103.71, 70.61, 70.54, 68.84, 68.51, 58.88, 58.82, 52.01; HR MS(ESI)m/z: calcd for C_29_H_29_O_4_N_6_Br_2_Na [M + Na]^+^683.0612, found 683.0624.

[6,7-Bis-(2-methoxy-ethoxy)-quinazolin-4-yl]-{3-[1-(3-methoxy-phenyl)-1H-[1,2,3]triazol-4-yl]-phenyl}-amine (4e). m.p.85-88°C; ^1^H NMR (600 MHz, DMSO-d_6_): *δ* 9.61 (s, 1H), 8.66 (s, 1H), 8.27 (s, 1H), 8.01 (s, 1H), 7.91 (d, *J* = 7.9 Hz, 1H), 7.57 (d, *J* = 7.6 Hz, 1H), 7.46 (t, *J* = 7.9 Hz, 1H), 7.32 (t, *J* = 7.9 Hz, 2H), 6.97 (s, 1H), 6.93 (d, *J* = 7.9 Hz, 2H), 5.63 (s, 2H), 4.32 (d, *J* = 9.1 Hz, 4H), 3.80 (d, *J* = 9.0 Hz, 4H), 3.76 (s, 3H), 3.39 (s, 3H), 3.37 (s, 3H).^13^C NMR (150Hz, DMSO-d_6_): 159.96, 156.75, 154.04, 148.63, 147.09, 140.46, 137.91, 131.41, 130.47, 129.50, 122.35, 122.13, 120.87, 120.51, 119.31, 114.27, 113.98, 108.94, 103.82, 87.75, 70.60, 70.54, 68.85, 68.54, 58.88, 58.83, 55.61, 53.46, 22.56; HR MS (ESI) m/z: calcd for C_30_H_33_O_5_N_6_ [M + H]^+^ 557.2512, found 557.2508.

[6,7-Bis-(2-methoxy-ethoxy)-quinazolin-4-yl]-{3-[1-(2-fluoro-phenyl)-1H-[1,2,3]triazol-4-yl]-phenyl}-amine (4f). m.p.83-86°C; ^1^H NMR (600 MHz, DMSO-d_6_): *δ* 9.62 (s, 1H), 9.11 (s, 1H), 8.51 (s, 1H), 8.39 (s, 1H), 8.04 – 7.86 (m, 3H), 7.73 – 7.59 (m, 3H), 7.56 – 7.46 (m, 2H), 7.25 (s, 1H), 4.37 – 4.28 (m, 4H), 3.83 – 3.74 (m, 4H), 3.39 (s, 3H), 3.37 (s, 3H). ^13^C NMR (150Hz, DMSO-d_6_): 156.85, 155.22, 154.08, 153.41, 148.57, 147.40, 140.64, 131.88, 130.76, 129.61, 126.56, 126.08, 123.40, 122.74, 121.08, 119.48, 117.75, 117.62, 109.46, 108.69, 103.71, 70.61, 70.54, 68.85, 68.51, 58.88, 58.82; HR MS (ESI) m/z: calcd for C_28_H_27_O_4_N_6_FNa [M + Na]^+^ 553.1970, found 553.1979.

[6,7-Bis-(2-methoxy-ethoxy)-quinazolin-4-yl]-{3-[1-(4-fluoro-phenyl)-1H-[1,2,3]triazol-4-yl]-phenyl}-amine (4g). m.p.88-91°C; ^1^H NMR (600 MHz, DMSO-d_6_): *δ* 9.73 (s, 1H), 9.32 (s, 1H), 8.57 (s, 1H), 8.38 (s, 1H), 8.14 – 7.86 (m, 4H), 7.67 (d, *J* = 7.6 Hz, 1H), 7.52 (dt, *J* = 12.5, 8.3 Hz, 3H), 7.26 (s, 1H), 4.41 – 4.25 (m, 4H), 3.85 – 3.72 (m, 4H), 3.39 (s, 3H), 3.37 (s, 3H); ^13^C NMR (150 Hz, DMSO-d_6_): 162.98, 161.35, 156.98, 156.96, 154.21, 153.11, 148.67, 147.80, 140.47, 133.71, 130.95, 129.63, 122.88, 122.82, 120.45, 119.61, 117.36, 117.20, 108.32, 103.79, 70.59, 70.53, 68.87, 68.57, 58.89, 58.83; HR MS (ESI) m/z: calcd for C_28_H_27_O_4_N_6_FNa [M + Na]^+^ 553.1970, found 553.1979.

[6,7-Bis-(2-methoxy-ethoxy)-quinazolin-4-yl]-{3-[1-(2-chloro-phenyl)-1H-[1,2,3]triazol-4-yl]-phenyl}-amine (4h). m.p.131-134°C; ^1^H NMR (600 MHz, DMSO-d_6_): *δ* 9.62 (s, 1H), 9.08 (s, 1H), 8.51 (s, 1H), 8.40 (s, 1H), 7.94 (d, *J* = 10.6 Hz, 2H), 7.85 – 7.79 (m, 2H), 7.70 – 7.61 (m, 3H), 7.52 (t, *J* = 7.9 Hz, 1H), 7.25 (s, 1H), 4.36 – 4.27 (m, 4H), 3.84 – 3.74 (m, 4H), 3.39 (s, 3H), 3.37 (s, 3H); ^13^C NMR (150Hz, DMSO-d_6_): 156.86, 154.09, 153.40, 148.58, 147.44, 146.99, 140.64, 135.03, 131.08, 130.89, 129.61, 129.13, 129.01, 128.96, 124.19, 122.67, 121.04, 119.48, 109.44, 108.66, 103.72, 70.61, 70.54, 68.85, 68.52, 58.88, 58.83; HR MS (ESI) m/z: calcd for C_28_H_27_O_4_N_6_ClNa [M + Na]^+^ 569.1675, found 569.1678.

[6,7-Bis-(2-methoxy-ethoxy)-quinazolin-4-yl]-{3-[1-(2-bromo-phenyl)-1H-[1,2,3]triazol-4-yl]-phenyl}-amine (4i). m.p.93-97°C; ^1^H NMR (600 MHz, DMSO-d_6_): *δ* 9.63 (s, 1H), 9.05 (s, 1H), 8.51 (s, 1H), 8.40 (s, 1H), 8.00 – 7.89 (m, 3H), 7.77 (dd, *J* = 7.8, 1.5 Hz, 1H), 7.67 (t, *J* = 7.7 Hz, 2H), 7.60 (t, *J* = 8.6 Hz, 1H), 7.52 (t, *J* = 7.9 Hz, 1H), 7.25 (s, 1H), 4.35 – 4.27 (m, 4H), 3.83 – 3.75 (m, 4H), 3.39 (s, 3H), 3.37 (s, 3H); ^13^C NMR (150Hz, DMSO-d_6_): 156.87, 154.10, 153.39, 148.58, 147.40, 146.92, 140.63, 136.72, 134.14, 132.58, 130.94, 129.61, 129.49, 129.24, 124.20, 122.64, 121.02, 119.47, 109.44, 108.64, 103.72, 100.00, 70.61, 70.54, 68.85, 68.52, 58.88, 58.83; HR MS (ESI) m/z: calcd for C_28_H_27_O_4_N_6_BrNa [M + Na]^+^ 613.1169, found 613.1180.

[6,7-Bis-(2-methoxy-ethoxy)-quinazolin-4-yl]-{3-[1-(4-bromo-phenyl)-1H-[1,2,3]triazol-4-yl]-phenyl}-amine (4j). m.p.105-108°C; ^1^H NMR (600 MHz, DMSO-d_6_): *δ* 9.63 (s, 1H), 9.37 (s, 1H), 8.51 (s, 1H), 8.38 (s, 1H), 7.96 (dd, J = 16.5, 7.6 Hz, 4H), 7.86 (d, *J* = 8.8 Hz, 2H), 7.66 (d, *J* = 7.6 Hz, 1H), 7.53 (t, *J* = 7.9 Hz, 1H), 7.25 (s, 1H), 4.32 (d, *J* = 24.8 Hz, 4H), 3.79 (d, *J* = 34.8 Hz, 4H), 3.39 (s, 3H), 3.37 (s, 3H); ^13^C NMR (150Hz, DMSO-d_6_): 156.86, 154.09, 153.40, 148.58, 147.97, 147.44, 140.63, 136.32, 133.32, 130.82, 129.62, 122.79, 122.38, 121.82, 121.01, 120.19, 119.48, 109.43, 108.66, 103.68, 70.60, 70.54, 68.84, 68.51, 58.88, 58.82; HR MS(ESI)m/z: calcd for C_28_H_27_O_4_N_6_BrNa [M + Na]^+^ 613.1169, found 613.1177.

[6,7-Bis-(2-methoxy-ethoxy)-quinazolin-4-yl]-{3-[1-(2-methoxy-phenyl)-1H-[1,2,3]triazol-4-yl]-phenyl}-amine (4k). m.p.87-90°C; ^1^H NMR (600 MHz, DMSO-d_6_): *δ* 9.62 (s, 1H), 8.94 (s, 1H), 8.50 (s, 1H), 8.36 (s, 1H), 7.98 – 7.91 (m, 2H), 7.69 (dd, J = 21.0, 7.7 Hz, 2H), 7.58 (t, *J* = 7.9 Hz, 1H), 7.50 (t, *J* = 7.9 Hz, 1H), 7.36 (d, *J* = 8.2 Hz, 1H), 7.25 (s, 1H), 7.19 (t, J = 7.6 Hz, 1H), 4.32 (d, *J* = 25.5 Hz, 4H), 3.90 (s, 3H), 3.79 (d, *J* = 34.6 Hz, 4H), 3.39 (s, 3H), 3.37 (s, 3H); ^13^C NMR (150Hz, DMSO-d_6_): 156.88, 154.08, 153.41, 152.32, 148.56, 147.45, 146.67, 140.57, 131.39, 131.21, 129.52, 126.43, 126.21, 123.94, 122.54, 121.36, 121.04, 119.43, 113.49, 109.45, 108.67, 103.73, 68.84, 68.51, 58.88, 58.52, 56.65; HR MS (ESI) m/z: calcd for C_29_H_30_O_5_N_6_Na [M + Na]^+^ 565.2170, found 565.2172.

[6,7-Bis-(2-methoxy-ethoxy)-quinazolin-4-yl]-[3-(1-*p*-tolyl-1H-[1,2,3]triazol-4-yl)-phenyl]-amine (4l). m.p.95-98°C; ^1^H NMR (600 MHz, DMSO-d_6_): *δ* 9.63 (s, 1H), 9.28 (s, 1H), 8.51 (s, 1H), 8.37 (s, 1H), 7.95 (d, *J* = 9.9 Hz, 2H), 7.87 (d, *J* = 8.3 Hz, 2H), 7.67 (d, *J* = 7.6 Hz, 1H), 7.52 (t, *J* = 7.9 Hz, 1H), 7.45 (d, *J* = 8.3 Hz, 2H), 7.25 (s, 1H), 4.32 (d, *J* = 25.1 Hz, 4H), 3.79 (d, *J* = 35.1 Hz, 4H), 3.39 (s, 3H), 3.37 (s, 3H), 2.51 (s, 3H); ^13^C NMR (150Hz, DMSO-d_6_): 156.88, 154.09, 153.40, 148.58, 147.70, 147.40, 140.59, 138.83, 134.89, 131.06, 130.75, 129.57, 122.69, 121.02, 120.36, 120.02, 119.46, 109.43, 108.64, 103.69, 70.60, 70.54, 68.84, 68.52, 58.88, 58.82, 21.07; HR MS (ESI) m/z: calcd for C_29_H_31_O_4_N_6_ [M + H]^+^ 527.2401, found 527.2410.

[6,7-Bis-(2-methoxy-ethoxy)-quinazolin-4-yl]-{3-[1-(3-nitro-phenyl)-1H-[1,2,3]triazol-4-yl]-phenyl}-amine (4m). m.p.98-101°C; ^1^H NMR (600 MHz, DMSO-d_6_): *δ* 9.64 (s, 1H), 9.60 (s, 1H), 8.83 (t, *J* = 2.1 Hz, 1H), 8.50 (d, *J* = 12.4 Hz, 2H), 8.41 (s, 1H), 8.37 (d, *J* = 7.5 Hz, 1H), 7.99 – 7.94 (m, 3H), 7.69 (d, *J* = 7.8 Hz, 1H), 7.55 (t, *J* = 7.9 Hz, 1H), 7.25 (s, 1H), 4.32 (d, *J* = 25.9 Hz, 4H), 3.79 (d, *J* = 36.0 Hz, 4H), 3.39 (s, 3H), 3.37 (s, 3H); ^13^C NMR (150Hz, DMSO-d_6_): 156.86, 154.09, 153.40, 149.06, 148.59, 147.45, 140.67, 137.70, 132.09, 130.64, 129.68, 126.40, 123.61, 122.92, 121.03, 120.61, 119.55, 115.08, 109.43, 108.66, 103.67, 70.61, 70.54, 68.84, 68.52, 58.88, 58.83; HR MS (ESI) m/z: calcd for C_28_H_27_O_6_N_7_Na [M + Na]^+^ 580.1915, found 580.1923.

[6,7-Bis-(2-methoxy-ethoxy)-quinazolin-4-yl]-{3-[1-(3-ethoxy-phenyl)-1H-[1,2,3]triazol-4-yl]-phenyl}-amine (4n). m.p.110-114°C; ^1^H NMR (600 MHz, DMSO-d_6_): *δ* 9.63 (s, 1H), 9.36 (s, 1H), 8.51 (s, 1H), 8.37 (s, 1H), 7.95 (d, *J* = 10.1 Hz, 2H), 7.67 (d, *J* = 7.7 Hz, 1H), 7.55 (d, *J* = 36.4 Hz, 4H), 7.25 (s, 1H), 7.08 (d, *J* = 10.2 Hz, 1H), 4.32 (d, *J* = 24.9 Hz, 4H), 4.17 (q, *J* = 7.0 Hz, 2H), 3.79 (d, *J* = 35.2 Hz, 4H), 3.39 (s, 3H), 3.37 (s, 3H), 1.39 (t, *J* = 7.0 Hz, 3H); ^13^C NMR (150Hz, DMSO-d_6_): 159.98, 156.87, 154.08, 153.42, 148.57, 147.84, 147.49, 140.61, 138.13, 131.37, 130.97, 129.59, 122.77, 121.01, 120.19, 119.48, 115.27, 112.27, 109.46, 108.69, 106.46, 103.68, 70.61, 70.54, 68.84, 68.51, 64.12, 58.88, 58.82, 15.02; HR MS (ESI) m/z: calcd for C_30_H_32_O_5_N_6_Na [M + Na]^+^ 579.2326, found 579.2332.

### Cell Antiproliferative Activity Assay

Cell antiproliferative activity was evaluated by the Cell Counting Kit-8 (CCK8, DOJINDO, Japan) assay. The cells were seeded at a density of 2,000 cells per well into 96-well microplate in 100 μl of growth medium. Cells were incubated at 37°C and 5% CO_2_ overnight. The next day, 100 μl per well of diluted inhibitor in growth medium was added with the final concentration from 0.1 nM to 100 μM. The cells were treated with DMSO as control. A series of dilutions are made in 0.1% DMSO in assay medium so that the final concentration of DMSO is 0.1% in all of treatments. Cells were incubated at 37°C and 5% CO_2_ for 48 h. Then, 10 μl of CCK8 was added to each well. The plates were incubated at 37°C for 2 h; after that, the plates were recorded by measuring absorbance at 450 nm with the reference wavelength of 630 nm using an EnVision Multilabel Reader (PerkinElmer). The IC_50_ values were calculated using GraphPad Prism 6.0 software and determined by the concentration causing a half-maximal percent activity. All assays were conducted with three parallel samples and three repetitions.

### Flow Cytometry Detection for Cell Apoptosis

Cell-apoptosis analysis was carried out by flow cytometry using the Annexin V/PI apoptosis kit (Solarbio, China) according to the manufacturer’s manual. Briefly, HeLa (5 × 10^4^/well) cells were seeded in 12-well plates for 24 h and then treated with 0.1% DMSO (as control) or various concentrations of compounds 4d, 4k, and 4l for 72 h, respectively. Cells were harvested, washed with PBS, and then incubated with 100 μl of 1 × Annexin V binding buffer containing 1 μl FITC Annexin V for 10 min at RT in the dark. Cells were incubated for another 5 min at room temperature (RT) in the dark after 1 μl PI was added. PBS (200 µl) was added to each tube for flow cytometry analysis (BriCyte E6). The percentages of apoptotic cells were analyzed using FlowJo soft.

### Flow Cytometry Detection for Cell Cycle

HeLa (5 × 10^4^/well) cells were seeded in 12-well plates for 24 h and then treated with 0.1% DMSO (as control), compounds 4d, 4k, and 4l of various concentrations for 48 h, respectively. The treated cells were harvested, washed with PBS, and then stained using the cell-cycle staining kit [Multisciences (Lianke) Biotech] according to the manufacturer’s manual. The distribution of cell-cycle phases with different DNA contents was determined by flow cytometry (BriCyte E6) and analyzed using ModFit LT software.

### Colony Formation Assay

HeLa cells were seeded in six-well plates at the density of 500/well for 24 h, and then treated with 0.1% DMSO (as control), various concentrations of compounds 4d, 4k, and 4l, respectively. The cells were incubated for 2 weeks in a 5% CO_2_ environment at 37°C for colony formation. The media was gently removed from each of the plates, and then each plate was washed with PBS twice. The colonies were fixed with 4% polyformaldehyde for 10 min and then wash the cells with ddH_2_O twice. Stain with 1 ml Crystal Violet Staining Solution (Beyotime, China) for 10 min. Wash excess crystal violet with ddH_2_O and allow dishes to dry. Take pictures of the plate and count the colonies.

### EGFR Kinase Assay

Kinase inhibitory activities of compounds were evaluated using the enzyme-linked immunosorbent assay (ELISA). The kinase enzyme of EGFR was purchased from Carna Bioscience (Kobe, Japan). A total of 10 ng/ml anti phosphotyrosine (PY713) antibody (abcam, Cambridge Science Park, United Kingdom) was precoated in 96-well ELISA plates. Active kinases were incubated with indicated drugs in one X reaction buffer (50 mmol/L HEPES pH 7.4, 20 mmol/L MgCl_2_, 0.1 mmol/L MnCl_2_, 1 mmol/L DTT) containing 20 μmol/L substrate (NH2-ETVYSEVRK-biotin) at 25°C for 1 h. Then, a total of 3 μmol/L ATP was added, and the reaction was continued for 2 h. The products of reaction were transferred into 96-well ELISA plates containing antibody and incubated at 25°C for 30 min. After incubation, the wells were washed with PBS and then incubated with horseradish peroxidase (HRP)-conjugated streptavidin. The wells were visualized using 3,3′,5,5′-tetramethylbenzidine (TMB), and chromogenic reaction was ended with 2 mol/L H_2_SO_4_, the absorbance was read with a multimode plate reader (PerkinElmer, United States) at 450 nm.

## Data Availability

The original contributions presented in the study are included in the article/Supplementary Material. Further inquiries can be directed to the corresponding authors.

## References

[B1] Akita R. W. Sliwkowski M. X. (2003). Preclinical Studies with Erlotinib (Tarceva). Semin. Oncol. 30, 15–24. 10.1016/s0093-7754(03)70011-6 12840797

[B2] Bhatia P. Sharma V. Alam O. Manaithiya A. Alam P. Kahksha (2020). Novel Quinazoline-Based EGFR Kinase Inhibitors: A Review Focussing on SAR and Molecular Docking Studies (2015-2019). Eur. J. Med. Chem. 204, 112640. 10.1016/j.ejmech.2020.112640 32739648

[B3] Bray F. Ferlay J. Soerjomataram I. Siegel R. L. Torre L. A. Jemal A. (2018). Global Cancer Statistics 2018: GLOBOCAN Estimates of Incidence and Mortality Worldwide for 36 Cancers in 185 Countries. CA Cancer J. Clin. 68, 394–424. 10.3322/caac.21492 30207593

[B4] Chavan P. V. Desai U. V. Wadgaonkar P. P. Tapase S. R. Kodam K. M. Choudhari A. (2019). Click Chemistry Based Multicomponent Approach in the Synthesis of Spirochromenocarbazole Tethered 1,2,3-triazoles as Potential Anticancer Agents. Bioorg. Chem. 85, 475–486. 10.1016/j.bioorg.2019.01.070 30776558

[B5] Cohen M. H. Williams G. A. Sridhara R. Chen G. Pazdur R. (2003). FDA Drug Approval Summary: Gefitinib (ZD1839) (Iressa) Tablets. Oncologist 8, 303–306. 10.1634/theoncologist.8-4-303 12897327

[B6] Hill E. K. (2020). Updates in Cervical Cancer Treatment. Clin. Obstet. Gynecol. 63 (1), 3–11. 10.1097/grf.0000000000000507 31815773

[B7] Hirsch F. R. Scagliotti G. V. Mulshine J. L. Kwon R. Curran W. J. Wu Y. L. (2017). Lung Cancer: Current Therapies and New Targeted Treatments. Lancet 389, 299–311. 10.1016/s0140-6736(16)30958-8 27574741

[B8] Hong V. Steinmetz N. F. Manchester M. Finn M. G. (2010). Labeling Live Cells by Copper-Catalyzed Alkyne-Aazide Click Chemistry. Bioconjug. Chem. 21, 1912–1916. 10.1021/bc100272z 20886827 PMC3014321

[B9] Jänne P. A. Yang J. C.-H. Kim D.-W. Planchard D. Ohe Y. Ramalingam S. S. (2015). AZD9291 in EGFR Inhibitor-Resistant Non-small-cell Lung Cancer. N. Engl. J. Med. 372, 1689–1699. 10.1056/NEJMoa1411817 25923549

[B10] Liontos M. Kyriazoglou A. Dimitriadis I. Dimopoulos M. A. Bamias A. (2019). Systemic Therapy in Cervical Cancer: 30 Years in Review. Crit. Rev. Oncol. Hematol. 137, 9–17. 10.1016/j.critrevonc.2019.02.009 31014518

[B11] Maddili S. K. Katla R. Kannekanti V. K. Bejjanki N. K. Tuniki B. Zhou C. H. (2018). Molecular Interaction of Novel Benzothiazolyl Triazolium Analogues with Calf Thymus DNA and HSA-Their Biological Investigation as Potent Antimicrobial Agents. Eur. J. Med. Chem. 150, 228–247. 10.1016/j.ejmech.2018.02.056 29529502

[B12] Mao L. Sun G. Zhao J. Xu G. Yuan M. Li Y. M. (2020a). Design, Synthesis and Antitumor Activity of Icotinib Derivatives. Bioorg. Chem. 105, 104421. 10.1016/j.bioorg.2020.104421 33181408

[B13] Mao L. F. Wang Y. W. Zhao J. Xu G. Q. Yao X. J. Li Y. M. (2020b). Discovery of Icotinib-1,2,3-Triazole Derivatives as Ido1 Inhibitors. Front. Pharmacol. 11, 579024. 10.3389/fphar.2020.579024 33101032 PMC7555427

[B14] Mathew M. P. Tan E. Saeui C. T. Bovonratwet P. Liu L. Bhattacharya R. (2015). Metabolic Glycoengineering Sensitizes Drug-Resistant Pancreatic Cancer Cells to Tyrosine Kinase Inhibitors Erlotinib and Gefitinib. Bioorg. Med. Chem. Lett. 25, 1223–1227. 10.1016/j.bmcl.2015.01.060 25690786 PMC5753412

[B15] Moyer J. D. Barbacci E. G. Iwata K. K. Arnold L. Boman B. Cunningham A. (1997). Induction of Apoptosis and Cell Cycle Arrest by CP-358,774, an Inhibitor of Epidermal Growth Factor Receptor Tyrosine Kinase. Cancer Res. 57, 4838–4848. 9354447

[B16] Qi Z. Y. Hao S. Y. Tian H. Z. Bian H. L. Hui L. Chen S. W. (2020). Synthesis and Biological Evaluation of 1-(benzofuran-3-Yl)-4-(3,4,5-Trimethoxyphenyl)-1h-1,2,3-Triazole Derivatives as Tubulin Polymerization Inhibitors. Bioorg. Chem. 94, 103392. 10.1016/j.bioorg.2019.103392 31669093

[B17] Qin X. Li Z. Yang L. Liu P. Hu L. Zeng C. (2016). Discovery of New [1,4]dioxino[2,3-F]quinazoline-Based Inhibitors of EGFR Including the T790M/L858R Mutant. Bioorg. Med. Chem. 24, 2871–2881. 10.1016/j.bmc.2016.01.003 27234887

[B18] Roskoski R. Jr. (2014). The ErbB/HER Family of Protein-Tyrosine Kinases and Cancer. Pharmacol. Res. 79, 34–74. 10.1016/j.phrs.2013.11.002 24269963

[B19] Saeedi M. Mohammadi-Khanaposhtani M. Pourrabia P. Razzaghi N. Ghadimi R. Imanparast S. (2019). Design and Synthesis of Novel Quinazolinone-1,2,3-Triazole Hybrids as New Anti-diabetic Agents: *In Vitro* α-glucosidase Inhibition, Kinetic, and Docking Study. Bioorg. Chem. 83, 161–169. 10.1016/j.bioorg.2018.10.023 30366316

[B20] Safavi M. Ashtari A. Khalili F. Mirfazli S. S. Saeedi M. Ardestani S. K. (2018). Novel Quinazolin-4(3h)-One Linked to 1,2,3-triazoles: Synthesis and Anticancer Activity. Chem. Biol. Drug Des. 92, 1373–1381. 10.1111/cbdd.13203 29637699

[B21] Schettino C. Bareschino M. A. Ricci V. Ciardiello F. (2008). Erlotinib: an EGF Receptor Tyrosine Kinase Inhibitor in Non-small-cell Lung Cancer Treatment. Expert Rev. Respir. Med. 2, 167–178. 10.1586/17476348.2.2.167 20477246

[B22] Tan C. S. Gilligan D. Pacey S. (2015). Treatment Approaches for EGFR-Inhibitor-Resistant Patients with Non-small-cell Lung Cancer. Lancet OncolOncology 16, e447–e459. 10.1016/S1470-2045(15)00246-6 26370354

[B23] Thomopoulou P. Sachs J. Teusch N. Mariappan A. Gopalakrishnan J. Schmalz H. G. (2015). New Colchicine-Derived Triazoles and Their Influence on Cytotoxicity and Microtubule Morphology. ACS Med. Chem. Lett. 7, 188–191. 10.1021/acsmedchemlett.5b00418 26985296 PMC4753549

[B24] Yin Y. Qiu X. Y. Zhang Y. H. Zhang B. (2019). A Rare Cutaneous Phototoxic Rash after Vandetanib Therapy in a Patient with Thyroid Cancer: A Case Report. Medicine (Baltimore) 98, e16392. 10.1097/MD.0000000000016392 31374006 PMC6709084

[B25] Zhang H. Q. Gong F. H. Ye J. Q. Zhang C. Yue X. H. Li C. G. (2017). Design and Discovery of 4-Anilinoquinazoline-Urea Derivatives as Dual TK Inhibitors of EGFR and VEGFR-2. Eur. J. Med. Chem. 125, 245–254. 10.1016/j.ejmech.2016.09.039 27688180

