# Supplementary figures and images for: Synthesis and Antitumor Activity of Erlotinib Derivatives Linked With 1,2,3-Triazole
Source: Front Pharmacol. 2022 Jan 17;12:793905. doi: 10.3389/fphar.2021.793905 (PMC8802806; doi:10.3389/fphar.2021.793905)

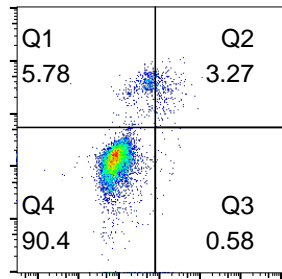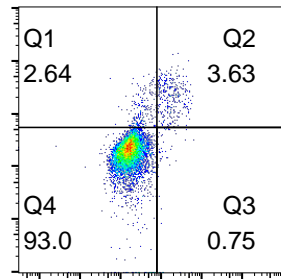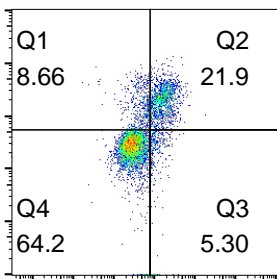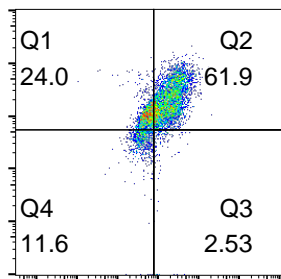

Supplement: Supplementary file 1 [file DataSheet3.zip › Apoptosis assay/Apoptosis 4k/Apoptosis Repeat 1/09-Oct-2021-Layout.pdf]

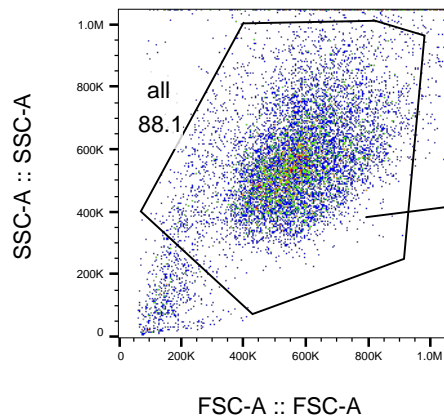

48h\_4n 3um\_2021\_09\_26\_16\_52\_30.fcs  
Ungated  
10000

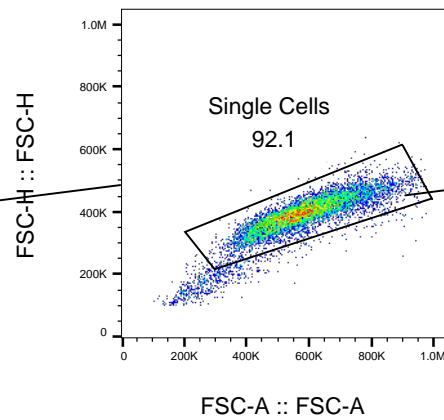

48h\_4n 3um\_2021\_09\_26\_16\_52\_30.fcs  
all  
8806

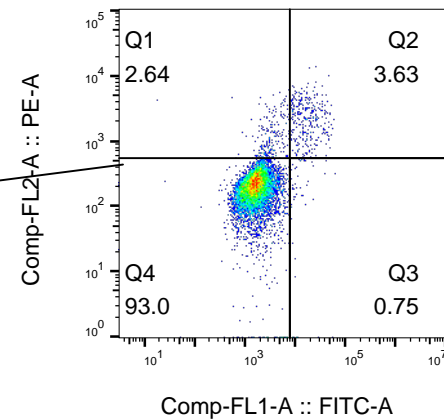

48h\_4n 3um\_2021\_09\_26\_16\_52\_30.fcs  
Single Cells  
8106

Supplement: Supplementary file 1 [file DataSheet3.zip › Apoptosis assay/Gating strategy fot apoptosis assay.pdf]

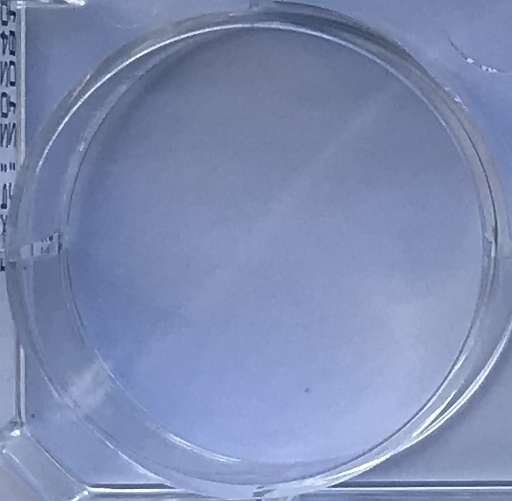

Supplement: Supplementary file 2 [file DataSheet4.zip › Colony formation assay/4d/Hela 4d 12 uM-1.jpg]

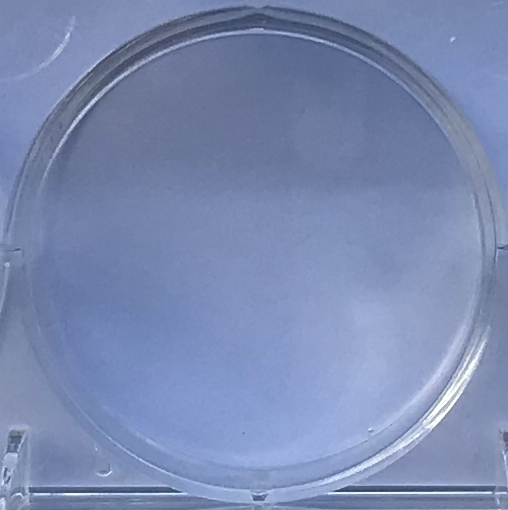

Supplement: Supplementary file 2 [file DataSheet4.zip › Colony formation assay/4d/Hela 4d 12 uM-2.jpg]

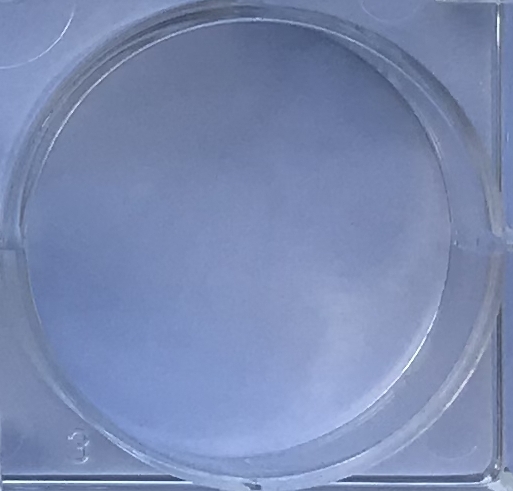

Supplement: Supplementary file 2 [file DataSheet4.zip › Colony formation assay/4d/Hela 4d 12 uM-3.jpg]

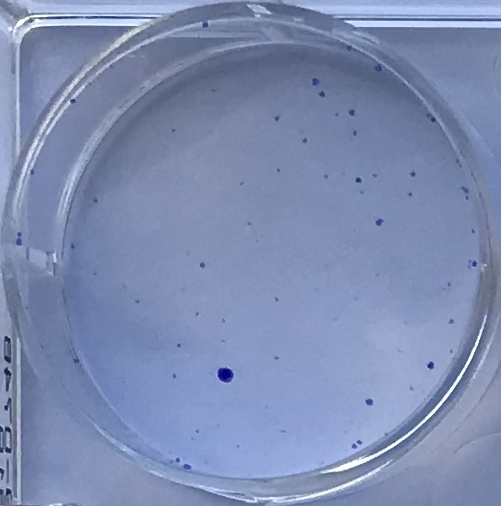

Supplement: Supplementary file 2 [file DataSheet4.zip › Colony formation assay/4d/Hela 4d 6 uM -1.jpg]

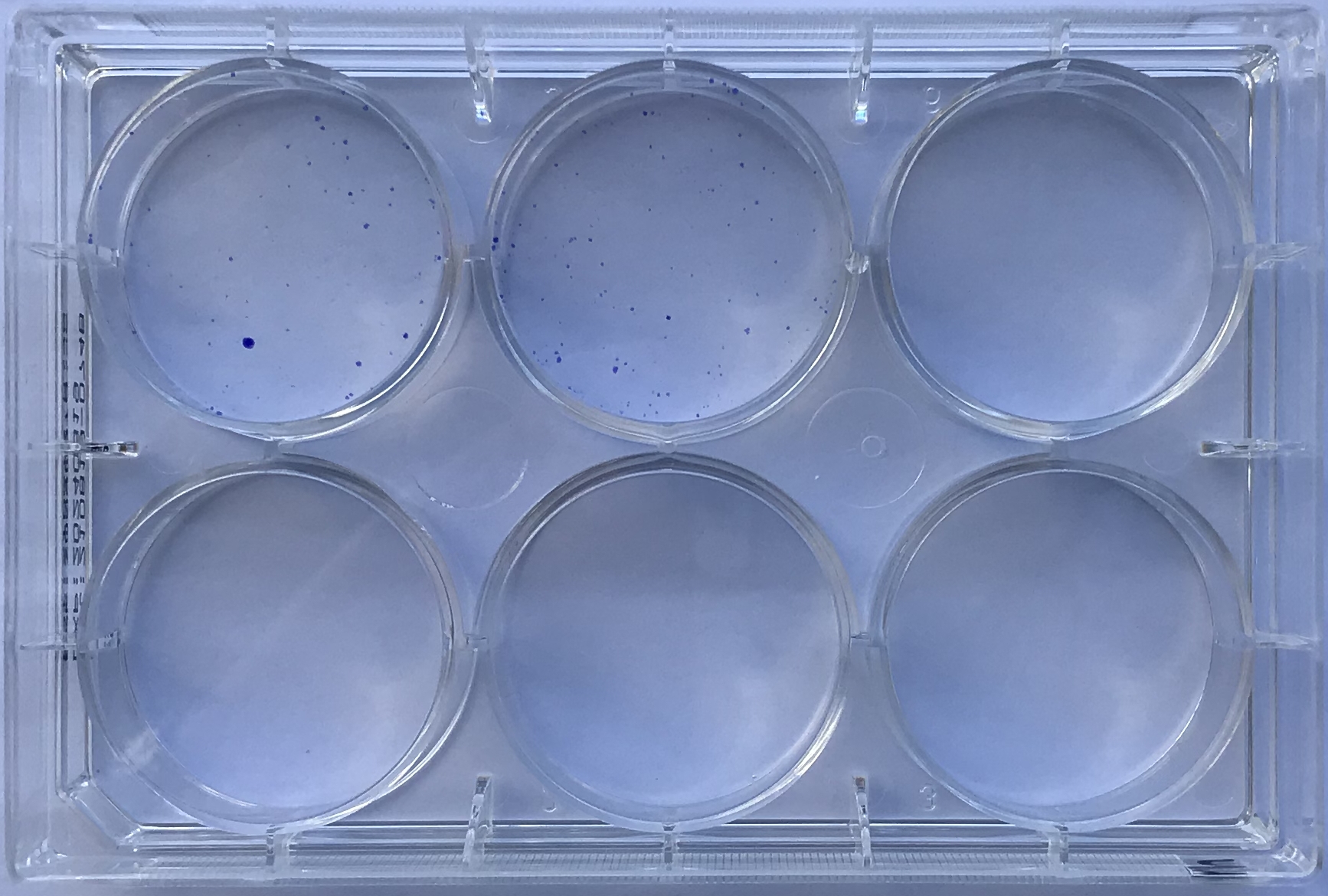

Supplement: Supplementary file 2 [file DataSheet4.zip › Colony formation assay/4d/Hela 4d 6 uM 12 uM.jpg]

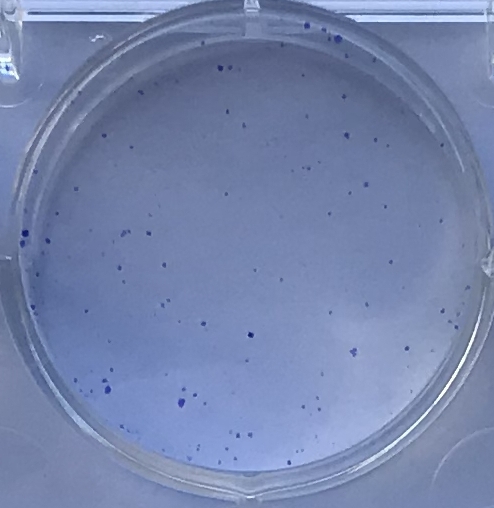

Supplement: Supplementary file 2 [file DataSheet4.zip › Colony formation assay/4d/Hela 4d 6 uM-2.jpg]

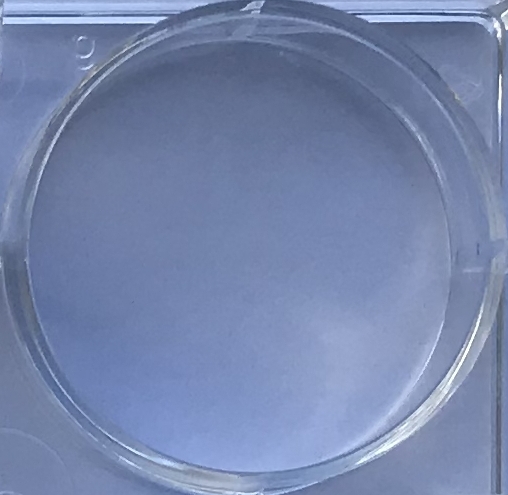

Supplement: Supplementary file 2 [file DataSheet4.zip › Colony formation assay/4d/Hela 4d 6 uM-3.jpg]

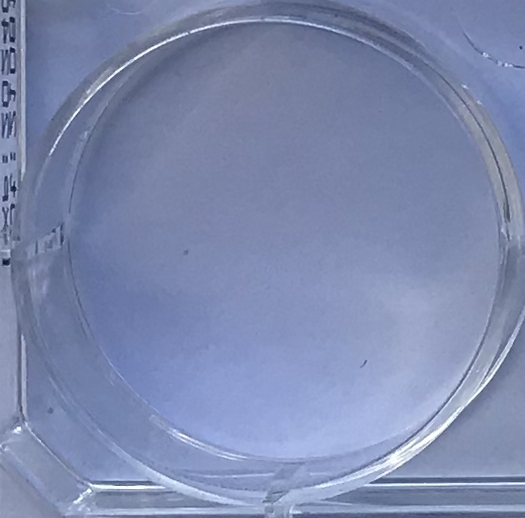

Supplement: Supplementary file 2 [file DataSheet4.zip › Colony formation assay/4l/Hela 4l 12uM-1.jpg]

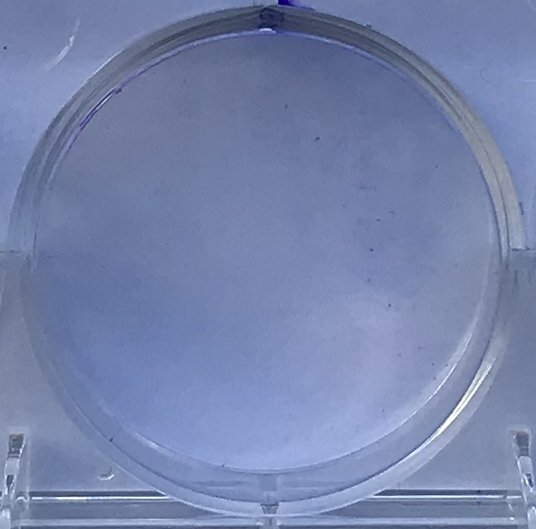

Supplement: Supplementary file 2 [file DataSheet4.zip › Colony formation assay/4l/Hela 4l 12uM-2.jpg]

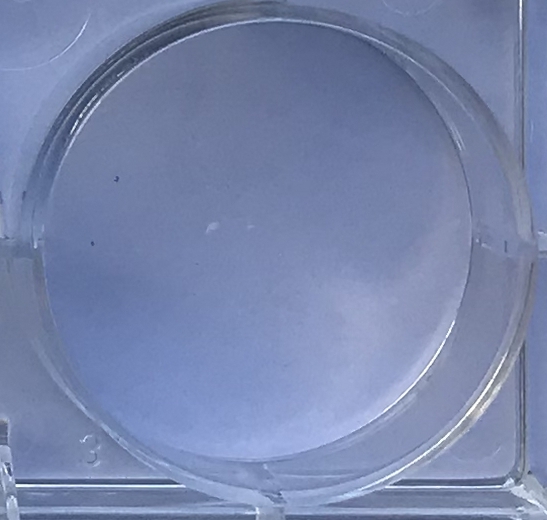

Supplement: Supplementary file 2 [file DataSheet4.zip › Colony formation assay/4l/Hela 4l 12uM-3.jpg]

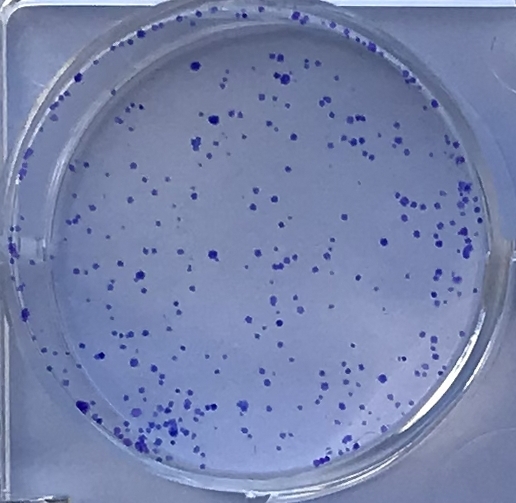

Supplement: Supplementary file 2 [file DataSheet4.zip › Colony formation assay/4l/Hela 4l 6uM -1.jpg]

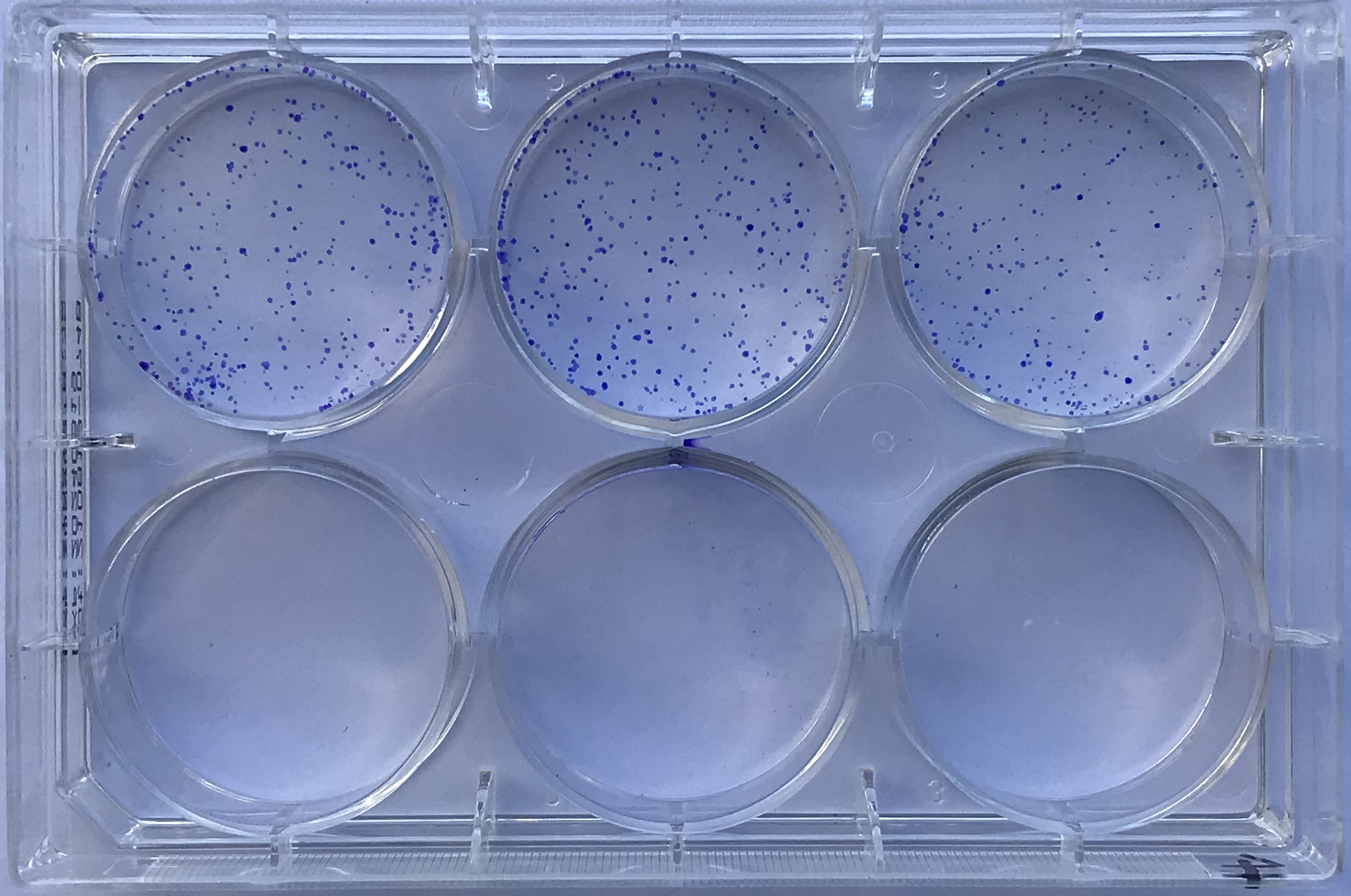

Supplement: Supplementary file 2 [file DataSheet4.zip › Colony formation assay/4l/Hela 4l 6uM 12uM.jpg]

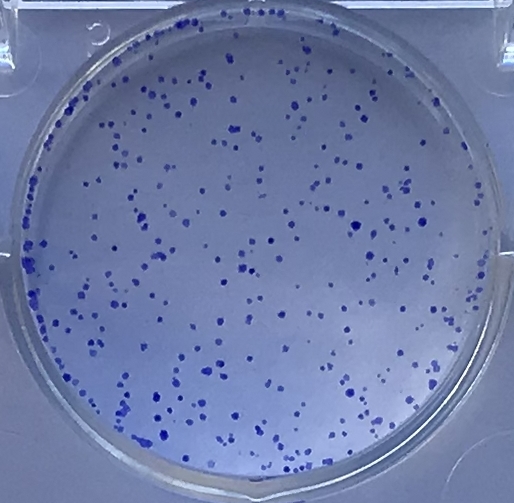

Supplement: Supplementary file 2 [file DataSheet4.zip › Colony formation assay/4l/Hela 4l 6uM-2.jpg]

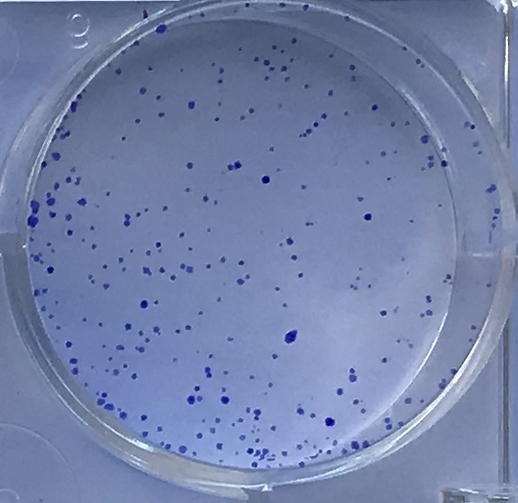

Supplement: Supplementary file 2 [file DataSheet4.zip › Colony formation assay/4l/Hela 4l 6uM-3.jpg]

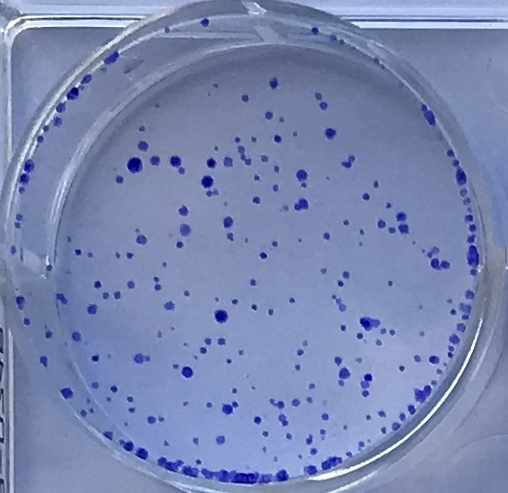

Supplement: Supplementary file 2 [file DataSheet4.zip › Colony formation assay/Hela NC-1.jpg]

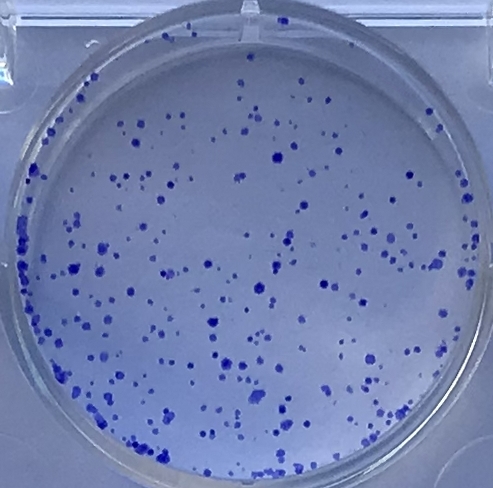

Supplement: Supplementary file 2 [file DataSheet4.zip › Colony formation assay/Hela NC-2.jpg]

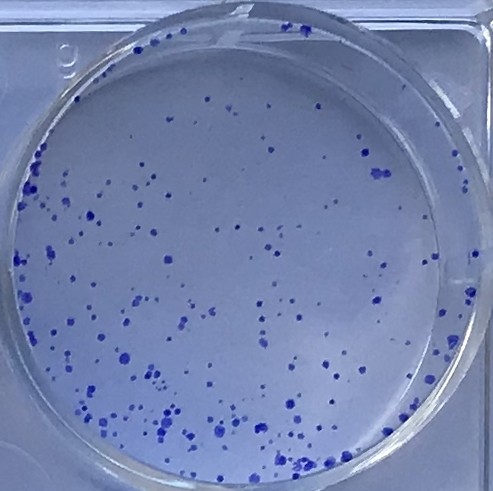

Supplement: Supplementary file 2 [file DataSheet4.zip › Colony formation assay/Hela NC-3.jpg]

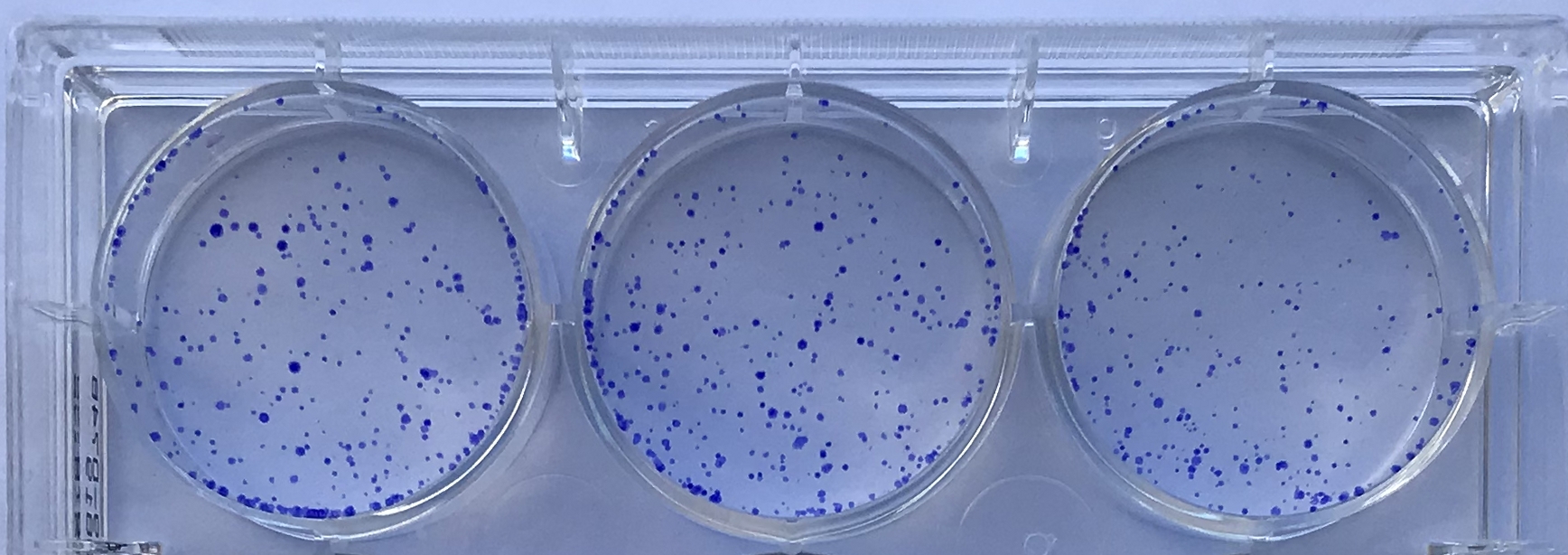

Supplement: Supplementary file 2 [file DataSheet4.zip › Colony formation assay/Hela NC.jpg]
