# Supplementary material for: Synthesis and Antitumor Activity of Erlotinib Derivatives Linked With 1,2,3-Triazole
Source: Front Pharmacol. 2022 Jan 17;12:793905. doi: 10.3389/fphar.2021.793905 (PMC8802806; doi:10.3389/fphar.2021.793905)
Supplement: Supplementary file 3 [file DataSheet1.docx]

# Supplementary Data

# Figure S1-1. ^1^H NMR spectrum (600MHz, DMSO-d_6_) of compound 4a

# Figure S1-2. ^13^C NMR spectrum (150MHz, DMSO-d_6_) of compound 4a

# Figure S1-3. HR MS of compound 4a

# Figure S2-1. ^1^H NMR spectrum (600MHz, DMSO-d_6_) of compound 4b

# Figure S2-2. ^13^C NMR spectrum (150MHz, DMSO-d_6_) of compound 4b

# Figure S2-3. HR MS of compound 4b

# Figure S3-1. ^1^H NMR spectrum (600MHz, DMSO-d_6_) of compound 4c

# Figure S3-2. ^13^C NMR spectrum (150MHz, DMSO-d_6_) of compound 4c

# Figure S3-3. HR MS of compound 4c

# Figure S4-1. ^1^H NMR spectrum (600MHz, DMSO-d_6_) of compound 4d

# Figure S4-2. ^13^C NMR spectrum (150MHz, DMSO-d_6_) of compound 4d

# Figure S4-3. HR MS of compound 4d

# Figure S5-1. ^1^H NMR spectrum (600MHz, DMSO-d_6_) of compound 4e

# Figure S5-2. ^13^C NMR spectrum (150MHz, DMSO-d_6_) of compound 4e

# Figure S5-3. HR MS of compound 4e

# Figure S6-1. ^1^H NMR spectrum (600MHz, DMSO-d_6_) of compound 4f

# Figure S6-2. ^13^C NMR spectrum (150MHz, DMSO-d_6_) of compound 4f

# Figure S6-3. HR MS of compound 4f

# Figure S7-1. ^1^H NMR spectrum (600MHz, DMSO-d_6_) of compound 4g

# Figure S7-2. ^13^C NMR spectrum (150MHz, DMSO-d_6_) of compound 4g

# Figure S7-3. HR MS of compound 4g

# Figure S8-1. ^1^H NMR spectrum (400MHz, DMSO-d_6_) of compound 4h

# Figure S8-2. ^13^C NMR spectrum (100MHz, DMSO-d_6_) of compound 4h

# Figure S8-3. HR MS of compound 4h

# Figure S9-1. ^1^H NMR spectrum (600MHz, DMSO-d_6_) of compound 4i

# Figure S9-2. ^12^C NMR spectrum (150MHz, DMSO-d_6_) of compound 4i

# Figure S9-3. HR MS of compound 4i

# Figure S10-1. ^1^H NMR spectrum (400MHz, DMSO-d_6_) of compound 4j

# Figure S10-2. ^13^C NMR spectrum (100MHz, DMSO-d_6_) of compound 4j

# Figure S10-3. HR MS of compound 4j

# Figure S11-1. ^1^H NMR spectrum (400MHz, DMSO-d_6_) of compound 4k

# Figure S11-2. ^13^C NMR spectrum (100MHz, DMSO-d_6_) of compound 4k

# Figure S11-3. HR MS of compound 4k

# Figure S12-1. ^1^H NMR spectrum (400MHz, DMSO-d_6_) of compound 4l

# Figure S12-2. ^13^C NMR spectrum (100MHz, DMSO-d_6_) of compound 4l

# Figure S12-3. HR MS of compound 4l

# Figure S13-1. ^1^H NMR spectrum (600MHz, DMSO-d_6_) of compound 4m

# Figure S13-2. ^13^C NMR spectrum (150MHz, DMSO-d_6_) of compound 4m

# Figure S13-3. HR MS of compound 4m

# Figure S14-1. ^1^H NMR spectrum (600MHz, DMSO-d_6_) of compound 4n

# Figure S14-2. ^13^C NMR spectrum (150MHz, DMSO-d_6_) of compound 4n

# Figure S14-3. HR MS of compound 4n
